# The ENDOPAIN 4D Questionnaire: A New Validated Tool for Assessing Pain in Endometriosis

**DOI:** 10.3390/jcm10153216

**Published:** 2021-07-21

**Authors:** Anne Puchar, Pierre Panel, Anne Oppenheimer, Joseph Du Cheyron, Xavier Fritel, Arnaud Fauconnier

**Affiliations:** 1 EA 7285 Research Unit ‘Risk and Safety in Clinical Medicine for Women and Perinatal Health’, Versailles-Saint-Quentin University (UVSQ), 78180 Montigny-le-Bretonneux, France; anneoppenheimer@yahoo.fr (A.O.); arnaud.fauconnier@ght-yvelinesnord.fr (A.F.); 2 Department of Gynecology and Obstetrics, Centre Hospitalier de Versailles, 78150 Le Chesnay, France; ppanel@ch-versailles.fr; 3 Department of Reproductive Medicine and Fertility Preservation, Hôpital Universitaire Antoine Béclère, 157, Rue de la Porte de Trivaux, 92140 Clamart, France; 4 Department of Gynecology and Obstetrics, Centre Hospitalier Intercommunal de Poissy-Saint-Germain-en-Laye, 78300 Poissy, France; joseph.ducheyron@ght-yvelinesnord.fr; 5 Department of Obstetrics and Gynaecology, University Hospital of Poitiers, 86000 Poitiers, France; xavier.fritel@univ-poitiers.fr

**Keywords:** endometriosis, questionnaires, pain assessment, pain symptoms in endometriosis, validation study

## Abstract

Objectives: To study the measurement properties, the responsiveness and the minimal clinically important difference of the ENDOPAIN-4D: a new questionnaire for assessing pain in endometriosis. Methods: A prospective, observational, multicentre study was conducted including all women ≥18 years consulting for symptomatic proven endometriosis between 1 January 2017 and 30 June 2018 and volunteering to participate. Each patient had to answer a new self-administered patient-reported outcome (PRO) questionnaires (the ENDOPAIN-4D) at inclusion (T0) and 12 months after medical or surgical treatment (T1). Criteria defined by COSMIN were used to validate the questionnaire’s measurement properties. The minimal clinically important difference was estimated by the anchor-based method. Results: The study included 199 women. The ENDOPAIN-4D score had a four dimensional structure with good internal consistency (measured by Cronbach α): (I) pain-related disability (α = 0.79), (II) painful bowel symptoms (α = 0.80), (III) dyspareunia (α = 0.83), and (IV) painful urinary tract symptoms (α = 0.77). They produced four subscores that can be summed to obtain a single score (α = 0.61). The ENDOPAIN-4D total score ranged from 0 to 94.00 (mean ± SD: 46.7 ± 22). The total score was significantly correlated with the PROs used in endometriosis. Sensitivity to change was good with large effect sizes (ES) (mean of the differences: 36.3 *p* = 1.8 10^−7^, ES 0.76). The minimal clinically important difference of the global score was determined to be 10.9. Conclusions: The ENDOPAIN-4D questionnaire is easy to use, valid, and effective in assessing patient reported pain symptoms in women treated for endometriosis. This new instrument can be used as the primary outcome for future clinical trials and as a tool for routine patient follow-up.

## 1. Introduction

Endometriosis is a chronic inflammatory disease that occurs in about 10% of women of childbearing age [1]. Up to 80% of these cases cause pain (associated with dysmenorrhoea, dyspareunia, pelvic pain, and bowel disorders) and impair quality of life [2], and women with endometriosis have an average quality of life 20% lower than that of healthy women [3].

Consideration of patients’ feelings about their symptoms and the impact of various diseases or symptoms on their quality of life has developed substantially in recent years with the increasing use of patient-reported outcomes (PROs), assessed by questionnaires or other tools [4]. PROs for assessing symptoms and quality of life are frequently used with patients with endometriosis to assess the potential benefits of treatment [3,5,6,7]. The literature includes various scales for pain assessment in endometriosis, most often visual analogue scales. Numerical and verbal rating scales are also frequently used [8,9]. These three types of scales make it possible to score pain, for example, from 0 to 10 (‘0’: no pain, ‘10’: worst pain) for each specific symptom. Nevertheless, they assess only the intensity of symptoms, without taking their heterogeneity into account.

To date, there is no score able to assess overall pain in endometriosis by considering all of its heterogeneous symptoms. For this reason, we have designed a self-administered questionnaire: the ENDOPAIN-4D questionnaire. Initially based on women’s verbal descriptions [10] to measure the pain symptoms of endometriosis, it was developed further through a modified DELPHI survey of patients and physicians [11]. The final questionnaire thus demonstrated content validity, that is, showed that it measured the subjective experiences of women with pain from endometriosis. Accordingly, it may provide a solid basis on which to develop an efficient and effective patient-centred instrument to measure these pain symptoms [11].

The translation was performed according to previously published guidelines [12]. Three native English-speaking women, bilingual in French, involved in the field of gynaecology translated the French version into an English draft version. Together, the translators consolidated their translations into a single first English version. Then, three French native bilingual health-care providers experienced in gynaecology, back-translated this first version into French (without having seen the original French version). The three initial translators then reviewed the three back-translation against the original English version, and provided a final forward translation.

The aim of this study is to assess the measurement properties of the ENDOPAIN-4D according to criteria defined by the COSMIN taxonomy, we also examined the responsiveness and determine its minimal clinically important difference.

## 2. Materials and Methods

### 2.1. ENDOPAIN-4D Development

**Figure 1:**
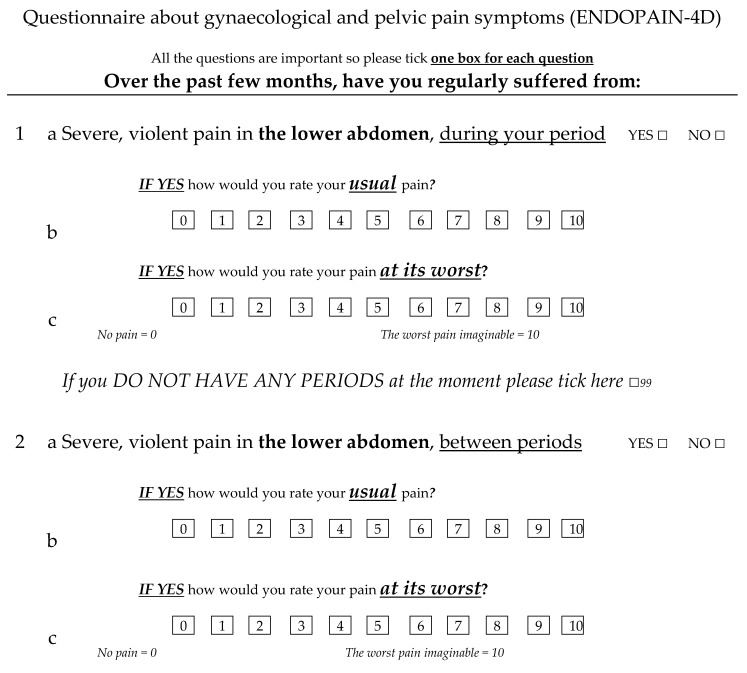
Extract from the ENDOPAIN-4D questionnaire.

The ENDOPAIN-4D comprises 21 items divided into four subparts: spontaneous pelvic pain and dysmenorrhoea (questions 1 to 10), dyspareunia (questions 11 to 13), intestinal pain symptoms (questions 14 to 16), and other symptoms (questions 17 to 21). Each question is first scored dichotomously as yes/no. If this answer is ‘yes’, the woman scores it according to the extent of the symptom from ‘0’: no pain to ‘10’: worst pain imaginable. If the answer is ‘no’, a score of ‘0’ is automatically assigned on the numerical rating scale. Ten questions relate to different types of pain (scored on two different numerical rating scale—one for usual pain and one for pain at its worst), and 11 cover discomfort or patients’ feeling about their pain symptoms (Figure 1; the entire questionnaire is available on demand).

### 2.2. Recruitment Sites and Treatment

This study took place at three French hospitals: Centre Hospitalier de Versailles, Centre Intercommunal de Poissy-Saint-Germain, and the University Hospital of Poitiers between 1 January 2017 and 30 June 2018. All three are reference centres for the management of endometriosis and use the same pretherapeutic approach: assessing each woman’s pain and quality of life by standardized self-administered questionnaires. All women underwent imaging as appropriate to their symptoms with at least an ultrasound (US) scan or magnetic resonance imaging (MRI) by practitioners experienced in endometriosis and working in a reference centre.

### 2.3. Endometriosis Treatment

Physicians and patients jointly decided on the option of medical or surgical management, with physicians deciding the type of hormonal treatment and its timing (prescribed before or after surgery). Medical treatment uses both analgesics and combined or progestogen-only hormonal contraceptive pills or a Gonadotropin-Releasing Hormone (GnRH) agonist. Surgical treatment consists, whenever possible, of complete excision of endometriosis lesions. The type of surgery depends on the lesion characteristics and locations [13].

### 2.4. Study Population

The study included all women aged 18 years or older consulting for symptomatic proven endometriosis in one of the three centres between 1 January 2017 and 30 June 2018 and volunteering to participate. For women with surgical management, proof came from histological results, and for those treated medically, by typical images of endometriosis implants (i.e., cysts and/or deep nodules) on radiological assessment. On US, lesions appeared as solid nodules of variable sizes, hypo- or iso-echoic, regardless of the regularity of their contours. They could also be in the form of hypoechoic thickening of the intestinal wall, vagina, or bladder [14]. On MRI, endometriosis was defined as a hypointense area and/or hyperintense foci on T1- or T2-weighted images, located mainly in the uterosacral ligaments, the torus uterinum, vagina, rectovaginal septum, rectosigmoid, pouch of Douglas, parametrium, bladder, and round ligaments [14].

Each included patient had to answer at least one of the ENDOPAIN-4D questions to be included in the analysis. The exclusion criteria were: no health insurance, inability to read French, presence of another major pelvic pathology, chronic pain other than endometriosis, and history of major pelvic surgery (including resection of deep endometriosis).

### 2.5. Design and Data Collection

At the first visit before treatment (T0), each women who agreed to participate in the study completed a self-administered questionnaire containing, alongside the ENDOPAIN-4D, epidemiologic data (age, gravidity, parity, and medical and surgical history) and different validated PROs widely used in the field of endometriosis and pelvic pathology: the French version of the International Consultation on Incontinence Questionnaire-Female Lower Urinary Tract Symptoms (ICIQ-Fluts), the International Prostate Symptom Score (IPSS) [15], the Knowles-Eccersley-Scott Symptoms Questionnaire (KESS) assessing bowel disorders, the Sexual Activity Questionnaire (SAQ) [16], the Endometriosis Health Profile 5 (EHP 5), and the EuroQOL 5D-3L (EQ5D-3L) [17].To assess the chief pain symptom, we also used a numerical rating scale (NRS) ranging from 0 ‘no pain’ to 10 ‘unbearable pain’ to assess pain ‘in the present tense’, ‘usual pain’ and ‘worst case pain’ during the last three months.

All women were asked to complete the same questionnaire 12 months (T1) after medical or surgical treatment. Simultaneously, they also rated their overall improvement of pain symptoms, with the Clinical Global Impression (CGI) scale-global improvement item [18].The CGI-I is a single-question: ‘how are your symptoms (pain and others) now compared with how they were before treatment?’ rated by seven answers: (i) ‘Much better’, (ii) ‘Better’, (iii) ‘Somewhat better’, (iv) ‘No change’, (v) ‘Somewhat worse’, (vi) ‘Worse’ and (vii) ‘Much worse’.

### 2.6. Statistical Analysis

In order to be used in clinical or research practice, any questionnaire need a complex and specific validation respecting the recommendations issued by the Consensus-based Standards for the selection of health Measurement Instruments (COSMIN).

We therefore used the COSMIN criteria [19] for the quantitative validation of this questionnaire. For items scored on two different scales (usual pain and pain at its worst), we decided to use the ’usual pain’ scales for the analysis. Descriptive statistics of the data, score distribution, and floor and ceiling effects (defined as >70% of respondents who responded by choosing the lowest or highest category respectively) were examined. A scree plot was constructed with factor analysis and component analysis with varimax rotation to examine the structural validity which measures the degree to which the scores of a health-related PRO instrument are an adequate reflection of the dimensionality of the construct to be measured. Internal consistency was assessed with Cronbach’s α, considered acceptable if >0.7 and good if >0.8.

Construct validity—which determines ‘how well a test measures what it is supposed to measure?’ by comparing the questionnaire to other questionnaires that measure similar qualities—was evaluated by testing the presupposed relations of the ENDOPAIN-4D total score and its four subscores with PROs used and validated in endometriosis as well as with prespecified characteristics and extent of disease. We therefore hypothesised a gradation of pain based on endometriosis severity according to its revised American Society for Reproductive Medicine (rASRM) stage [20], and on the presence of anterior or posterior endometriosis, the size of endometriomas, the number of locations (without endometriomas), and the obliteration of the pouch of Douglas [21].

Pearson correlation coefficients and analysis of variance (ANOVA) were used to test the relations between the ENDOPAIN-4D scores and the continuous and categorical variables, respectively.

For variables correlated with the ENDOPAIN-4D scores, we estimated the effect size according to Cohen’s method and its confidence interval. An effect size of 0.2 was considered a small effect, 0.5 a moderate effect, and 0.8 or higher a large effect.

For continuous variables, the median score was used as the cut-off to define groups.

Sensitivity or responsiveness to change was calculated by comparing results of the 4 subscores and the ENDOPAIN-4D total score between T1 and T0 with the paired data Student’s t-test. Effect sizes were then obtained by Cohen’s method.

The minimal clinically important difference (MCID) is the smallest difference perceived by patients as beneficial or harmful in the score of an instrument’s measure [22]. Because there is no ‘gold standard’ method for its estimation [23], we used an anchor-based method with the CGI scale-global improvement item. [18]

All statistical analyses were done with the R Studio version 4.0.3 software (2020-10-10) Copyright © 2021 The R Foundation for Statistical Computing Platform. 51 Franklin Street, Fifth Floor, Boston, MA 02110-1301, USA.

### 2.7. Ethics

Our study was purely observational and involved no intervention. As such, no written informed consent was required by the relevant statute, the Huriet-Serusclat Act dated 20 December 1998. Nevertheless, all patients received information about the study and were free to participate or not. The confidentiality of patients’ data has been respected. The IVth southeast Ethics Committee (Sud-Est, n° 18/002) in France approved this study.

## 3. Results

### Participants

**Figure 2:**
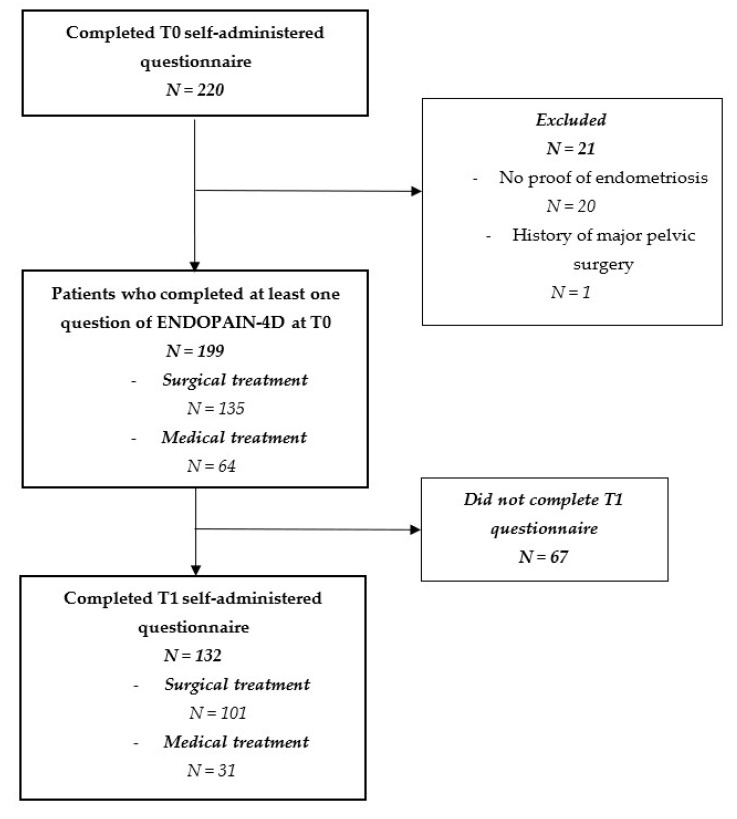
Flow chart of the validation study of the ENDOPAIN-4D questionnaire, women who completed the questionnaire at inclusion (T0) and those who completed it at one year follow-up (T1).

The study included 199 women who completed the T0 questionnaire (Figure 2). Among them, 132 (66.3%) completed the self-administered questionnaire at T1. Table 1 lists the characteristics of patients at baseline and T1. The 132 women who completed the ENDOPAIN-4D questionnaire at T1 did not differ significantly from the 67 who did not (Table 1).

The descriptive analyses of the ENDOPAIN-4D questionnaire at T0 are presented in Table S1 (Supplementary Materials). Missing data was <5% for all variables except for ‘pain before period’ (13.6%), ‘dyspareunia’, ‘positional dyspareunia’, and ‘interruption of sexual intercourse’ (13.1, 13.1, and 11.6% respectively).

A floor effect was observed for ‘right shoulder pain’, which was thus excluded from further analysis. 

The question on infertility was excluded from further analysis because it did not relate to a painful symptom.

The scree plot showed that the ENDOPAIN-4D questionnaire was multidimensional (data not shown). Principal component analysis with orthogonal varimax rotation found a four-dimensional structure (Table 2) with good internal consistency: I) pain-related disability (Cronbach α = 0.79), II) painful bowel symptoms (Cronbach α = 0.80), III) dyspareunia (Cronbach α = 0.83), and iv) painful urinary tract symptoms (α = 0.77) resulting in four subscores (pain-related disability, painful bowel symptoms, dyspareunia, painful urinary tract symptoms).

As the first dimension, which included three of the 19 questionnaire items, accounted for 17% of the total variance, we chose to sum it to obtain a single score (α = 0.63) The ENDOPAIN-4D total scores ranged from 0 to 94.00 (mean ± SD: 46.7 ± 22) for a maximum theoretical value of 110, where 0 corresponds to no pain and 110 to the worst pain possible.

Table S2 reports the Pearson correlation of the four ENDOPAIN-4D subscores and its total score with the PROs used in endometriosis. The total score was significantly correlated with all of the PROs validated for endometriosis that we used.

The ENDOPAIN-4D total score was significantly associated with the size of the endometriomas (≤3 cm versus >3 cm: *p* = 0.035, ES = 1.35). The score was not, on the other hand, significantly associated with the severity of endometriosis assessed by the rASRM stage, the presence of anterior or posterior DIE, the number of locations, or the obliteration of the pouch of Douglas.

Dyspareunia and functional urinary symptom subscores were significantly associated with the severity of endometriosis assessed by the rASRM stage (respectively, *p* = 0.029, ES = 0.5 and *p* = 0.02, ES = 0.5). No other significant association was found between the four subscores and the pre-established hypotheses.

**Figure 3:**
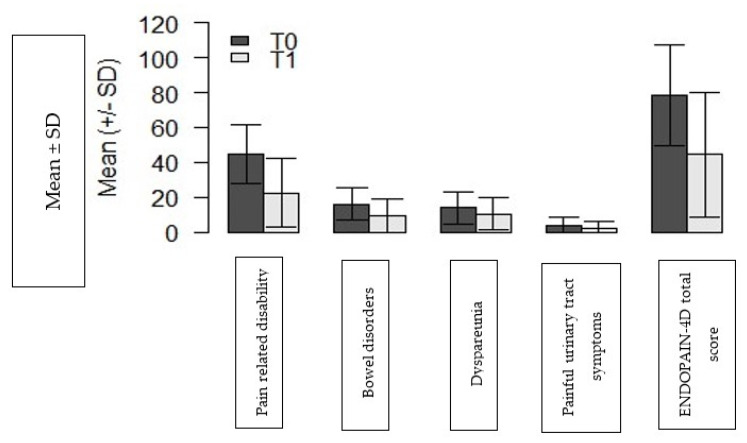
Responsiveness of the ENDOPAIN-4D questionnaire evaluating each subscore (pain related-disability, painful bowel symptoms, dyspareunia, painful urinary tract symptoms) and the ENDOPAIN-4D total score before (T0) and after treatment (T1). *n* = 132 patients who responded at T1.

The 132 patients who completed the ENDOPAIN-4D questionnaire at T0 and T1 comprise the population used to analyse responsiveness to change and to calculate the MCID. All four subscales differed significantly between T0 and T1 (Figure 3). Similarly, responsiveness was statistically significant for all subscores (pain related disability: *p* = 7.6 × 10*^−^*^9^; painful bowel symptoms: *p* = 7.1 × 10*^−^*^12^, dyspareunia *p* = 1.8 × 10*^−^*^4^ and painful urinary tract symptoms *p* = 1.1 × 10*^−^*^5^).

All results are statistically significant.

The ENDOPAIN-4D total score also decreased significantly after treatment (mean of the differences: 36.3, *p* = 1.8 × 10^−7^, ES 0.76) (Figure 3). Changes in scores between T0 and T1 were significantly associated with the woman’s response to CGI-I for each subscore and the ENDOPAIN-4D global score (*p* < 0.05). Of the 132 respondents at T1, 105 (79.5%) reported improvement on the CGI-I after treatment, and among these 105 patients, 24 had a difference of only one degree on CGI-I after treatment. This method showed that MCID for the ENDOPAIN-4D total score was 10.9.

## 4. Discussion

This prospective study provides evidence of the validity of the ENDOPAIN-4D questionnaire according to the COSMIN criteria for assessing pain in patients with endometriosis. The ENDOPAIN-4D was four-dimensional and thus made it possible to explore separately specific important domains related to endometriotic pain, including pain-related disability, sexual dysfunction, and bowel and lower urinary tract symptoms. Furthermore, adding together the four subscores produced a single score that captures the overall pain of endometriosis patients, regardless of the specific pain symptoms they have. The significant correlation between the ENDOPAIN-4D total score and the PROs generally used in endometriosis demonstrates the questionnaire’s construct validity. Finally, the changes in the ENDOPAIN-4D total score with surgical and medical treatment show its responsiveness to treatment.

### 4.1. Strengths and Weaknesses

The strength of this study lies in its methodological design, which meets all the criteria for questionnaire validation set forth by COSMIN. In addition, it is a prospective multicentric study including women with different subtypes of endometriotic involvement (deep, superficial, or both, with different locations) that was specifically designed to evaluate the psychometric and clinimetric properties of the ENDOPAIN-4D questionnaire.

Several limitations must nonetheless be emphasised:

First, the response rate for each item was acceptable except for items related to sexual intercourse. This disparity may be explained by the study’s inclusion of women without partners or by the intrusiveness of such questions, or both.

Second, it is a selected population. The recruitment of women at reference centres for endometriosis resulted in a high proportion of patients with severe endometriosis (37.4% with stage IV). Nevertheless, there are no clear design rules for sampling a target population to validate a health measurement instrument. For example, Terwee et al. recommended simply an adequate description of the target population to be able to judge the completeness and applicability of the questionnaire in other population [24].

Moreover, only weak associations were found between the extent of the disease, its locations, and the ENDOPAIN-4D total score. These data should not affect the validation of this questionnaire because many studies have failed to establish a link between some symptoms of endometriosis and lesion sites. The fact that the patients included in the study had severe forms of endometriosis and also severe symptoms, which lead a decision to treat, may result in referral bias [25], which in turn could explain our inability to relate the severity of pain symptoms to the variables measuring the extent of the disease. Nonetheless, this study demonstrated that the severity of the disease, evaluated by the rASRM score, was significantly associated with the dyspareunia and painful urinary tract symptom subscores, according to data published in several studies [26,27,28].

Although each of the subscores as well as the ENDOPAIN-4D total score are significantly correlated with the EHP-5, the EQ-5D 3L and EQ5D-VAS taking into account the psychological impact of endometriosis on patients; it is important to emphasize that no subscore of our questionnaire is entirely dedicated to the assessment of the negative impact of endometriosis on women’s psychological health and general wellbeing. To date, many studies have reported the impact of endometriosis on anxiety and depression [2,29] and recently, Arena et al. have underlined the importance of creating a trustful relationship with women in order to reduce their anxiety levels. [30]

Recent studies have reported the importance of superficial dyspareunia as an endometriosis-related symptom, defined as complaint of pain or discomfort on vaginal entry or at the vaginal introitus [31,32]. In a unicentric observational study on endometriosis, Mabrouk et al. reported 67.5% of superficial dyspareunia, concomitant with deep dyspareunia in more than 83% of cases. Only 18 out of the 160 patients (11.3%) had exclusive superficial dyspareunia. As reported by Kumar and Robertson [33], the causes of superficial dyspareunia are multiple: hormonal, infectious, inflammatory, dermatoses, neurologic and traumatic. In patients with endometriosis, several of these causes could therefore be intertwined (hormonal, traumatic and neurologic).’ Otherwise, our questionnaire was developed using a two-round modified DELPHI procedure mixing endometriosis patients and physicians to select a set of statements to describe the painful symptoms of endometriosis. [11] Superficial dyspareunia was not found as a painful symptom in this process. 

Finally, despite a correct response rate 12 months after treatment (T1) (66.3%) might raise the question of the risk of selection bias in the women who responded and could therefore call the results into question. We are reassured by the absence of significant differences between women who responded at follow-up and those who did not. Finally, the number of women included in the responsiveness study was sufficient to estimate the responsiveness and the MCID with precision.

To the best of our knowledge, our questionnaire is the only one to assess all the pain symptoms in endometriosis and to have been fully validated according to the COSMIN criteria. Only a few questionnaires assessing pain symptoms in endometriosis have been published, and most of them have been used to predict endometriotic involvement based on symptoms [31,32,33,34]. These scales have obvious interesting clinimetric properties for endometriosis assessment but they have not been evaluated in accordance with the methods recommended for developing PROs [35].

Two other scores have been developed to assess pain symptoms of endometriosis. The daily electronic Endometriosis Pain and Bleeding Diary, developed by Deal et al., is a self-administered questionnaire with 17 items that assess intermittent and continuous pain in endometriosis with good internal consistency. Although it appears able to characterize the types of pain that endometriosis patients identified as important, its use appears limited by the absence of studies of its dimensionality, construct validity, and responsiveness [36,37].

In 2018, Wyrwich et al. developed the Endometriosis Daily Pain Impact Diary Items to assess dysmenorrhoeal and non-menstrual pelvic pain [38]. This questionnaire derived from the daily electronic Endometriosis Pain and Bleeding Diary questionnaire described above has been fully validated; the properties of reliability, convergent validity, and responsiveness of the dysmenorrhoeal and non-menstrual pelvic pain daily items were examined quantitatively in a phase II clinical trial of an investigational treatment for endometriosis. This questionnaire does not, however, consider bowel and urinary tract disorders, as ours does. In addition, it is difficult to use because it requires daily measurements. Furthermore, in its current form, it does not provide for the calculation of a single score.

### 4.2. Implications

Previously published trials on pain treatment in endometriosis have lacked a single outcome capable of assessing all the various heterogenous pain symptoms of endometriosis patients [39,40]. The Biberoglu and Behrman score [41] combines the woman’s self-evaluation of three distinct symptoms (dysmenorrhoeal and pelvic pain and dyspareunia) and two physician observations during clinical examination (pelvic tenderness and induration). Each symptom is scored from 0 to 3 for ’absent’, ’mild’, ’moderate’, or ’severe’. Each of the subscores obtained by individual items is summed to form a total score. It has been used as the primary outcome for pain assessment in several trials [42]. The combination in a single score of symptoms and the clinical examination creates a high risk of bias and a lack of reproducibility. Inversely the ENDOPAIN-4D scale, as a self-administered measurement, overcomes this difficulty. It proved to be easy to use, valid and responsive to change. It is the first questionnaire fully patient-derived and validated (including responsiveness and MCID calculation) that assesses pain symptoms in endometriosis; it thus meets the specifications desired for a good primary outcome for pain trials [43]. Furthermore it can be used as a single (summed) assessment or separate pain symptom assessments, which makes it possible to assess the efficacy of therapy separately for specific symptoms (i.e., dyspareunia) and for the overall course of endometriosis. The ENDOPAIN-4D scale is easy, quick to complete, and inexpensive. Its requirement of only paper and pencil facilitates its use in the routine follow-up of women.

## 5. Conclusions

Because endometriosis is a common disease responsible for pain in 80% of cases and impairs quality of life, often severely, it is crucial to develop easy-to-use validated tools for clinical practice and therapeutic evaluation. The ENDOPAIN-4D PRO can be used as a primary outcome for future clinical studies but also as a tool for routine patient follow-up. An international multicentric evaluation outside the French setting is needed.

## Figures and Tables

**Table 1:** Baseline characteristics of women, overall and according to responses to the ENDOPAIN-4D questionnaire at T0 and T1.

	All Patients *n* = 199	Patients Who Completed ENDOPAIN-4D at T1 *n* = 132	Patients Who Did Not Complete ENDOPAIN-4D at T1 *n* = 67	*p* Value
Center *n* (%)				
- Versailles - Poissy - Poitiers	102 (51.0)	65 (49.2)	37 (55.2)	
	66 (33.0)	47 (35.6)	19 (28.4)	0.20
	31 (16.0)	20 (15.2)	11 (16.4)	
Main indications for endometriosis treatment *n* (%)				
- Pain - Infertility - Ovarian cysts - Other (bleeding)	180 (90.5)	118 (89.4)	62 (92.5)	
	17 (8.5)	13 (9.8)	4 (6.0)	0.20
	1 (0.5)	1 (0.8)	0 (0)	
	1 (0.5)	0	1 (1.5)	
Age—years mean (sd)	33.4 (6.8)	34.9 (7.2)	33.4 (7.7)	0.17
BMI—kg/m^2^ mean (sd)	24.0 (5.2)	24.7 (4.7)	23.1 (4.5)	0.04
Gravidity—mean (sd)	0.9 (1.32)	0.8 (1.3)	1.0 (1.4)	0.32
Parity—mean (sd)	0.6 (0.9)	0.5 (0.9)	0.7 (1.0)	0.47
Medical treatment only—*n* (%)	55 (27.6)	27 (20.5)	28 (41.8)	
Medical treatment and ART *n* (%)	9 (4.6)	4 (0.03)	5 (7.5)	0.20
Surgical treatment—*n* (%)	135 (67.8)	101(76.5)	34 (50.7)	
Usual pain (NRS)—mean (SD)	5.1 (1.6)	5.0 (2.4)	5.3 (2.2)	0.45
Worst case pain (NRS)—mean (SD)	8.6 (1.6)	8.6 (1.6)	8.7 (1.5)	0.77
rASRM stage *				
- I-Minimal—*n* (%) - II-Mild (%) - III-Moderate—*n* (%) - IV-Severe—*n* (%)	28 (21.9)	22 (23.2)	6 (9.0)	
	28 (21.9)	21 (22.1)	7 (10.4)	0.24
	24 (18.8)	16 (16.8)	8 (11.9)	
	48 (37.4)	36 (37.9)	12 (17.9)	
Anterior DIE **				
- None—*n* (%) - Uterine serosa—*n* (%) - Bladder—*n* (%)	123 (69.9)	83 (65.4)	40 (63.5)	0.21
	45 (25.6)	33 (26.0)	12 (19.0)	
	8 (4.5	7 (5.5)	1 (1.5)	
Posterior DIE **				
- None—*n* (%) - Uterosacral ligaments—*n* (%) - Vagina—*n* (%) - Vagina & rectosigmoid—*n* (%) - Rectosigmoid—*n* (%)	16 (9.0)	10 (7.9)	6 (9.2)	0.22
	100 (56.2)	72 (56.7)	28 (43.1)	
	8 (4.5)	6 (4.7)	2 (3.1)	
	4 (2.2)	3 (2.4)	1 (1.5)	
	50 (28.1)	32 (25.2)	18 (27.7)	

BMI: Body mass index, ART: Assisted reproductive technology, NRS: Numerical rating scale, SD: standard deviation, rASRM: Revised American Society for Reproductive Medicine Stage, DIE: Deeply infiltrating endometriosis, * For 135 patients with surgical treatment. Two patients had only parietal endometriosis, for five patients AFS stage is not available, ** For all patients: classification based on surgical results for surgical treatment and on imaging results for medical treatment. DIE classification according to DIE Location (Chapron et al., 2001), For anterior DIE: data are missing for 23 patients, for posterior DIE: data are missing for 21 patients.

**Table 2:** Factor analysis loadings of the items of ENDOPAIN-4D questionnaire from component analysis with varimax rotation. Nineteen items were used in the analysis. The item ’right shoulder pain’ was excluded because it had a floor effect. Results of factor analysis with an orthogonal rotation method with 4 factors. The correlation ≥0.4 of an item with a dimension appears in bold because the item is considered a component of the dimension.

Item (Condensed)	Pain-Related Disability	Painful Bowel Symptoms	Dyspareunia	Painful Urinary Tract Symptoms
*Dysmenorrhoea*	0.40	0.27	0.14	0.11
Non-menstrual pelvic pain	0.15	0.43	0.30	−0.01
Intense pain	0.68	0.14	0.18	0.19
Worsening pain	0.44	0.12	0.27	0.27
Pain before period	0.41	0.13	0.19	0.23
Stabbing pain	0.49	0.19	0.14	0.17
Lower back pain	0.30	0.17	0.15	0.14
Leg/hip pain	0.44	0.32	−0.07	0.05
Disabling pain	**0.78**	0.10	0.01	0.05
Pain affects mobility	**0.71**	0.06	0.07	−0.01
Dyspareunia	0.05	0.25	**0.79**	0.13
Positional dyspareunia	0.22	0.15	**0.68**	0.02
Interruption of sexual intercourse	0.11	−0.02	**0.76**	0.21
Painful bowel movements	0.20	**0.82**	0.10	0.05
Bowel spasms	0.13	**0.79**	0.10	0.05
Diarrhea/constipation	0.27	**0.55**	0.04	0.16
Pain when urinating	0.17	0.06	0.07	**0.75**
Bladder pain	0.21	0.16	0.24	**0.80**
Sciatica	0.35	0.26	0.05	0.08

## Data Availability

The data presented in this study are available on request from the corresponding author.

## Supplementary Materials

The following are available online at https://www.mdpi.com/article/10.3390/jcm10153216/s1. Table S1: Descriptive statistics and scoring distribution of the 20 items of the ENDOPAIN-4D questionnaire among endo-metriosis patients (Each item is scored on a scale from 0 [No pain] to 10 [Worst pain]), Table S2: Construct validity: Pearson correlation of the four subscores and the ENDOPAIN-4D total score with other patient-reported outcome instruments used for endometriosis patients.
