# Supplementary material for: The ENDOPAIN 4D Questionnaire: A New Validated Tool for Assessing Pain in Endometriosis
Source: J Clin Med. 2021 Jul 21;10(15):3216. doi: 10.3390/jcm10153216 (PMC8348422; doi:10.3390/jcm10153216)
Supplement: Supplementary file 1 [file jcm-10-03216-s001.zip › jcm-1263459-supplementary.pdf]

Supplementary Materials

Table S1. Descriptive statistics and scoring distribution of the 20 items of the ENDOPAIN-4D questionnaire among endometriosis patients (Each item is scored on a scale from 0 [No pain] to 10 [Worst pain]).

Item (condensed)	*Min*	*Q1*	*Median*	*Mean*	*Q3*	*Max*	*Missing data N (%)*	*SD*	*Skewness*
Dysmenorrhoea	0.0	4.0	6.0	5.9	8.0	10.0	3(1.5)	2.5	−0.74
Non-menstrual pelvic pain	0.0	0.0	4.0	3.9	6.0	10.0	7(3.5)	2.9	0.00
Intense pain	0.0	6.0	8.0	6.9	10.0	10.0	4(2.0)	3.6	−1.13
Worsening pain	0.0	0.0	8.0	6.0	9.0	10.0	6(3.0)	4.0	−0.61
Pain before period	0.0	2.0	6.0	5.5	8.0	10.0	27(13.6)	3.6	−0.43
Stabbing pain	0.0	4.0	8.0	6.2	9.0	10.0	10(5.0)	3.8	−0.81
Lower back pain	0.0	0.0	7.0	5.4	9.0	10.0	6(3.0)	3.8	−0.39
Leg/hip pain	0.0	0.0	2.0	3.6	8.0	10.0	5(2.5)	3.9	0.37
Disabling pain	0.0	3.0	6.0	5.3	8.0	10.0	2(1.0)	3.4	−0.49
Pain affects mobility	0.0	1.5	6.0	5.3	8.0	10.0	4(2.0)	3.6	−0.41
Dyspareunia	0.0	0.0	4.0	3.8	7.0	10.0	26(13.1)	3.2	0.11
Positional dyspareunia	0.0	3.0	6.0	5.6	8.0	10.0	26(13.1)	3.4	−0.49
Interruption of sexual intercourse	0.0	0.0	6.0	4.7	8.0	10.0	23(11.6)	4.0	−0.09
Painful bowel movements	0.0	0.0	4.0	3.8	7.0	10.0	5(2.5)	3.3	0.11
Bowel spasms	0.0	0.0	5.0	4.1	7.0	10.0	9(4.5)	3.1	−0.10
Diarrhoea/constipation	0.0	0.0	4.0	4.0	7.0	10.0	6(3.0)	3.1	−0.08
Pain when urinating	0.0	0.0	0.0	1.7	3.0	10.0	6(3.0)	2.7	1.41
Bladder pain	0.0	0.0	0.0	2.4	5.0	10.0	6(3.0)	2.9	0.73
Sciatica	0.0	0.0	0.0	1.9	4.0	10.0	8(4.0)	2.8	1.10
Right shoulder pain	0.0	0.0	0.0	2.7	7.0	10.0	8(4.0)	3.7	1.85

Min: minimum; Q1: 1st quartile; Q3: 3rd quartile; Max: maximum; SD: standard deviation.

Table S2. Construct validity: Pearson correlation of the four subscores and the ENDOPAIN-4D total score with other patient-reported outcome instruments used for endometriosis patients.

	**Pain-related disability**	**Painful bowel symptoms**	**Dyspareunia**	**Painful urinary tract symptoms**	**ENDO-PAIN-4D total score**
SAQ	**−0.21***	**−0.17***	**−0.48*****	−0.13	**−0.38*****
EHP-5	**0.66*****	**0.50****	**0.60*****	**0.41****	**0.75*****
EQ5D-3L	**−0.43*****	**−0.25*****	**−0.28*****	**−0.22****	**−0.40*****
EQ5D-VAS	**−0.37*****	**−0.16***	**−0.29*****	**−0.23****	**−0.40*****
KESS	**0.22****	**0.54*****	**0.22***	**0.17***	**0.41*****
IPSS	**0.24****	**0.29*****	**0.33*****	**0.60*****	**0.49*****
Worst case pain NRS	**0.48*****	**0.42*****	**0.39*****	**0.28*****	**0.57*****

*<0.05 **<0.01 ***<0.001, SAQ: Sexual activity questionnaire; EHP-5: Endometriosis Health Profile 5; EQ5D 3L: EuroQoL 5D-3L; EQ5D VAS: EuroQoL Visual Analogue Scale, KESS: Knowles-Eccersley-Scott Symptoms Questionnaire; IPSS: International Prostate Symptom Score, NRS: Numerical Rating Scale.
